# Comparative Assessment of Fungal Biofilm Development on PEEK and PDMS Biomaterials and the Antifungal Potential of TiO_2_/ZrO_2_-Coated PEEK

**DOI:** 10.3390/ma19194161

**Published:** 2026-09-29

**Authors:** Gabriela Angeles-de Paz, Luisa Ixchel Perez-Garcia, Fernando Torres-Ariza, Michelle Ramírez-Mireles, Hugo Martínez-Gutiérrez, Martín Daniel Trejo-Valdez, Aída Verónica Rodríguez-Tovar, Christopher René Torres-SanMiguel

**Affiliations:** 1 Instituto Politécnico Nacional, Escuela Superior de Ingeniería Mecánica y Eléctrica, Sección de Estudios de Posgrado e Investigación, Zacatenco, Mexico City 07300, Mexico; gangelesd@ipn.mx; 2 Instituto Politécnico Nacional, Escuela Nacional de Ciencias Biológicas, Departamento de Microbiología, Laboratorio de Micología Médica, Unidad Profesional Lázaro Cárdenas, Casco de Sto. Tomás, Mexico City 011340, Mexico; lperezg1700@alumno.ipn.mx (L.I.P.-G.); ftorresa2500@alumno.ipn.mx (F.T.-A.); mramirez1805@alumno.ipn.mx (M.R.-M.); 3 Instituto Politécnico Nacional, Centro de Nanociencias y Micro y Nanotecnologías (CNMN), Laboratorio de Microscopía de Barrido de Ultra Alta Resolución, Av. Luis Enrique Erro S/N, Unidad Profesional Adolfo López Mateos, Zacatenco, Gustavo A. Madero, Mexico City 07738, Mexico; humartinez@ipn.mx; 4 Instituto Politécnico Nacional-ESIQIE, Laboratorio de Investigación en Nanomateriales y Energías Limpias, Zacatenco, Gustavo A. Madero, Mexico City 07738, Mexico; mtrejov@ipn.mx

**Keywords:** biomaterials, antifungal, biofilm

## Abstract

**Highlights:**

- PDMS supported denser fungal biofilm than PEEK.
- TiO_2_/ZrO_2_ coatings reduced biofilm structures on biomedical materials.
- A thick TiO_2_-coated PEEK inhibited both fungal species’ biofilms in vitro maturation.
- Surface modification may improve the antifungal performance of coated PEEK.

**Abstract:**

Polyether ether ketones (PEEK) and polydimethylsiloxanes (PDMS) are widely used biomaterials; however, their susceptibility to microbial colonization remains a limitation in clinical applications. This study comparatively evaluated biofilm formation by *Candida albicans* and *Nakaseomyces glabratus* on PEEK and PDMS and characterized the effect of TiO_2_/ZrO_2_-based coatings applied to PEEK. Biofilm development was analyzed during adhesion, maturation, and dispersion using biomass quantification, metabolic activity assays, and scanning electron microscopy. Surface modifications were also evaluated. Both species formed a biofilm on the biomaterials, with a significantly higher biomass on the control, followed by PEEK and PDMS. TiO_2_/ZrO_2_-based coating affected biofilm maturation, with coating B as the one producing the greatest reduction in biofilm development. These findings suggest that surface modifications, such as hydrophobicity, using TiO_2_/ZrO_2_ coatings may influence fungal biofilm reduction and improve the antimicrobial performance of biomedical materials.

## 1. Introduction

In recent decades, the use of biomedical devices, such as intravascular catheters, prostheses, heart valves, and other implants, has increased significantly, improving patients’ quality of life. Indeed, the biocompatibility features of materials (biomaterials) ensure the safety of medical devices by assessing their compatibility with biological systems and their potential to cause harm or adverse reactions [1]. These materials are manufactured to support tissues and cell proliferation while avoiding toxicity. For instance, polymers such as polydimethylsiloxane (PDMS) and polyether ether ketone (PEEK) are widely used due to their favorable physicochemical properties and biocompatibility. PDMS, comprising silicon–oxygen bonds with methyl side groups, exhibits flexibility, hydrophobicity, thermal stability, and low toxicity, making it suitable for implants, prostheses, and catheters [2]. Conversely, PEEK is a semi-crystalline polyaromatic thermoplastic with high mechanical strength, chemical and thermal stability, and an elastic modulus comparable to that of bone, rendering it ideal for orthopedic, dental, and other implantable applications [3].

However, conventional biomaterials such as PEEK and PDMS do not prevent microbial adhesion *per se*. Therefore, strategies to reduce colonization and biofilm-associated infections have increasingly focused on surface modifications and antimicrobial coatings [4,5]. These approaches aim to preserve the favorable mechanical and biocompatible characteristics of the underlying biomaterial while introducing surface properties that may limit microbial adhesion, maturity and persistence. In this context, metal nanoparticle-based coatings have attracted attention as potential antimicrobial surface modifications for biomaterials [6,7]. Nevertheless, the efficacy of these treatments may range and vary according to the physicochemical specific biomaterial characteristics and the microorganisms involved. Indeed, the lack of these modifications and the hydrophobic nature of untreated biomaterials makes them susceptible to microbial adhesion and subsequent biofilm formation, thereby increasing the risk of infection, which has led to clinical concerns like high morbidity, mortality, and associated healthcare costs [8].

Most of these infections are currently classified as Healthcare-Associated Infections (HAIs), defined by the World Health Organization (WHO) as infections acquired during the provision of healthcare that may manifest during or after hospitalization. According to Asokan et al. [9], medical devices represent an important reservoir for biofilm-associated infections and HAIs due to their persistence and treatment resistance thanks to the production of extracellular matrix, which acts as a physical and chemical barrier against antimicrobial agents and the host immune response [10]. Biofilms are structured communities of microorganisms adhered to an either abiotic or biotic surface that exhibit significant phenotypic alterations, including reduced growth rates and increased tolerance to environmental stressors compared to their planktonic counterparts. In general, microbial biofilm development follows several stages, including adherence/attachment, proliferation, maturation, and dispersion. During this process, microorganisms modify hydrophobicity of surfaces, leading to an irreversible microbial attachment, then exopolysaccharide is synthesized using quorum sensing to enable coordinated growth and environmental adaptation, and finally, after biofilm maturation, microbes can be dispersed to continue their life cycle in a new substrate [11].

The most frequent microorganisms associated with medical biofilm infections include bacteria (*Escherichia coli*, *Klebsiella pneumoniae* and so on) and fungi (mainly *Candida* species) [12]. Among them, bacteria biofilms are extensively studied and normally results are manageable with antibiotics, whilst fungi biofilms may require device removal or a unique therapeutic and diagnosis [13]. Species of *Candida* are especially important given their common habitat on healthcare professionals’ skin, representing a potential source of horizontal transmission in the nosocomial setting [14]. These yeasts encompass a wide range of species, with *Candida albicans* and *Nakaseomyces glabratus* (formerly *Candida glabrata*) being the most prevalent and extensively studied species of both localized and systemic candidiasis and can even be part of a mix of biofilm-associated diseases [11]. Infections associated with them are closely linked to diverse medical devices, such as intravascular and urinary catheters, orthopedic implants, prostheses, cardiovascular devices, ventilators, and so on [15].

Although several studies have successfully incorporated biomaterials on pilot and used devices, none of them have considered fungal infection susceptibility via equipment management or horizontal transmission [16,17,18]. Thus, the biomaterials laboratory and medical mycology research team from the Instituto Politécnico Nacional had previously developed several PEEK interspinous spacers that were subjected to fungi colonization by *C. albicans*, which had revealed a specific susceptibility of biofilm infection of this material [19]. Based on these results, in 2024, Cabrera Rodríguez et al. [20] proposed TiO_2_/ZrO_2_ as an antimicrobial coating for steel samples and found efficient bactericidal activity against *K. pneumoniae* and *K. oxytoca*. Therefore, this study aimed to evaluate the ability of *C. albicans* and *N. glabratus* to form biofilms on PEEK and PDMS biomaterials and to determine the potentially antifungal effects of two different TiO_2_/ZrO_2_-coated PEEK on fungal biofilm development.

## 2. Materials and Methods

### 2.1. Chemicals and Culture Media

For fungi cultures, Yeast Peptone Dextrose, YPD, was prepared as a broth (1 g L^−1^ yeast extract, MCD Lab, Tultitlán, Mexico, 2 g L^−1^ peptone, MCD Lab, Tultitlán, Mexico, 2 g L^−1^ dextrose, DIBICO^®^, Cuatitlán, Mexico) and solid medium (18 g L^−1^ agar, MCD Lab, Tultitlán, Mexico). The medium Roswell Park Memorial Institute 1640 (RPMI) was purchased from Gibco^®^ (Thermo Fisher Scientific, Waltham, MA, USA) and supplemented with 2 g L^−1^ dextrose (DIBICO^®^, Cuatitlán, Mexico) and 4-(2-hydroxyethyl)-1-piperazineethanesulfonic acid (MOPS) buffer (Bio Basic, Markham, ON, Canada). Phosphate-buffered saline 1× (PBS) consisted of 8 g L^−1^ NaCl (JT Baker, Phillipsburg, NJ, USA), 0.2 g L^−1^ KCl (TQA S.A, Buenos Aires, Argentina), 1.44 g L^−1^ Na_2_HPO_4_ (JT Baker, Phillipsburg, NJ, USA), and 0.24 g L^−1^ KH_2_PO_4_ (JT Baker, Phillipsburg, NJ, USA) at pH = 7.4. MTS tetrazolium, 3-(4,5-dimethylthiazol-2-yl)-5-(3-carboxymethoxyphenyl)-2-(4-sulfophenyl)-2H-tetrazolium (Invitrogen^®^, Waltham, MA, USA) stock solution was prepared by dissolving it in 1× PBS to a final concentration of 0.5 mg mL^−1^. The solution was sterilized by filtration through a 0.22 µm pore-size membrane filter and stored at 4 °C until use.

### 2.2. Fungal Strains and Inoculum Preparation

The reference strains *Candida albicans* ATCC 10231 (American Type Culture Collection) and *Nakaseomyces glabratus* (formerly *Candida glabrata*) CBS 138 (Centraalbureau voor Schimmelcultures collection) were cultured in YPD broth at 37 °C and 170 rpm for 12 h. Blastoconidia were counted and adjusted at 1 × 10^6^ cells mL^−1^ in a Neubauer chamber (Qualvidrios Ltd.a, Campina Grande, Brazil).

### 2.3. Biomedical Material Production, Treatment, and Coating Preparation

PEEK material was selected for use in a 3D printer capable of reaching temperatures of 380–420 °C, as this is its melting point. Then, a slicer (computer program) was used to convert the 3D design into a layer-by-layer format, thereby setting the print parameters, such as a maximum speed of 60 mm/s, while the process is maintained in a moisture-free environment. When using a 0.4 mm nozzle, there is a risk of creating gaps where particles can accumulate, so a smooth surface is created to largely prevent these defects. Once the part is printed, it is allowed to cool for 30 to 60 min to ensure it does not warm up due to high temperatures.

PDMS involves a step-by-step process that allows the desired geometries to be obtained as follows. First, the design is established, and a master mold is printed, which can be printed of PLA to facilitate rapid polymerization due to its porosity. The solution consists of two components, A and B, which are mixed in a 10:1 ratio to achieve properties as close as possible to the ideal properties of PDMS. Once mixed, it is degassed in the open air or in a vacuum chamber, taking care not to spill the fluid. It is then poured into the mold, and there are two options at this point to cure the fluid: after 24 to 72 h in the open air, taking care to prevent it from becoming contaminated with particles such as dust, or an oven or hot plate can be used to accelerate the process—at 60 °C, this takes between 40 and 90 min, depending on the thickness of the material.

The TiO_2_/ZrO_2_-coated PEEK was prepared as described in Cabrera Rodríguez et al. [20]. The titanium–zirconium sol–gel solution was prepared as follows. For this study, titania sol–gel solutions were prepared with zirconium mole percentage of 5%, using zirconium (IV) propoxide (C_12_H_28_O_4_Zr, Sigma Aldrich, Burlington, MA, USA, 70%) as the zirconium precursor. First, as described in the previous section, titanium isopropoxide was dissolved in absolute ethanol. In a separate vessel, the acid hydrolysis of zirconium propoxide was performed by slowly adding it dropwise to a stirred mixture of absolute ethanol, nitric acid, and deionized water to adjust the final pH to 1.25 and achieve a water/[C_12_H_28_O_4_Zr + Ti (OC_3_ H_7_)_4_] molar ratio of 0.16. After one hour, the ethanolic solution of titanium isopropoxide was added to the vessel containing the pre-hydrolyzed zirconium propoxide mixture while maintaining stirring. The resulting sol–gel solutions were stored in sealed flasks and used to process the materials the following day.

For the synthesis of the films, flat substrates of PEEK PDMS, with diameters of 20 mm and thicknesses of 1 mm, were used. The flat surfaces of the substrates were rinsed with an aqueous solution prepared with 5% *v*/*v* of Extran MA 05 and rinsed with tap water. Then, the substrates were rinsed with distilled water and ethanol before drying with a nitrogen stream. The cleaned substrates were coated with the A or B sol–gel solutions described above using a home-built spin-coating device, operated under ambient conditions. A volume of 70 µL of the sol–gel solution was applied to the flat surfaces of the 316L steel disks, which were spun at 3000 rpm. To ensure the formation of homogeneous xerogel films, this coating process was repeated three times.

### 2.4. Experimental Design

Biofilm formation was evaluated using both *C. albicans* ATCC 10231 and *N. glabrata* CBS 138 strains after being cultured and adjusted to 1 × 10^6^ cells mL^−1^. Polyether ether ketone (PEEK) and polydimethylsiloxane (PDMS) disks were used as biomaterial substrates. Prior to experimentation, all disks were washed with neutral detergent and distilled water, autoclaved at 121 °C (15 psi) for 15 min, and exposed to ultraviolet (UV) irradiation for 30 min each side to ensure sterility. Sterilization validity was tested by incubating them at 37 °C for 48 h in RPMI 1640 medium. Biofilm formation assays were performed in 96- and 12-well plates by incubating the standardized inoculum in RPMI 1640 medium and supplemented with glucose and MOPS buffer at 37 °C for up to 72 h. Biofilm development was assessed at adhesion (4 h), maturation (24 h), and dispersion (48 h) stages using a destructive sampling design. Non-adherent fungus (yeast) was removed by washing with phosphate-buffered saline (PBS), and fresh medium was added for the later stages. In vitro biofilms were subjected to different analyses, including biomass quantification by crystal violet staining (Section 2.6) and metabolic activity using the MTS reduction determination (Section 2.7), while biofilm architecture and surface morphology were analyzed by scanning electron microscopy (SEM) (Section 2.8).

For the PEEK coating treatment, new PEEK disks were subjected to surface modification via TiO_2_ sol–gel coating, and the effect on fungal biofilm formation was qualitatively explored through SEM (Section 2.7). A control with a sol–gel matrix was also included. The surface modifications, including particle size, elemental analyses, and water angle contact, were additionally performed (Section 2.9).

Data were analyzed separately for *C. albicans* and *N. glabratus*. For biomass and metabolic activity analyses, the evaluated factors were the biomaterial surfaces (PS, PEEK, and PDMS) and biofilm developmental stages (adhesion, maturation, and dispersion). Because a destructive sampling design was employed, different disks were used for each biofilm developmental stage. Three replicate disks per experimental condition (technical replicates) were analyzed using a two-way analysis of variance (ANOVA) to evaluate the effects of the biomaterial surface and biofilm developmental stage, as well as their interaction. When significant differences were detected, multiple comparisons were performed using Tukey’s post hoc test. Prior to analysis, the assumptions of normality and homogeneity of variance were evaluated using the Shapiro–Wilk and Brown–Forsythe tests, respectively. Statistical analyses were performed using SigmaPlot version 15.1 (Grafiti LLC, Palo Alto, CA, USA), and differences were considered statistically significant at *p* < 0.05.

### 2.5. In Vitro Biofilm Formation of C. albicans and N. glabratus

The in vitro biofilm formation assays (biofilm kinetics and biofilm assay on biomaterials) were performed as described by Thein et al. [21] and Camarillo-Márquez et al. [22], in 96- and 12-well flat-bottom plates (Corning, NY, USA). Briefly, each well was filled with a corresponding PEEK disk, 200 µL of each adjusted inoculum, and 2.4 mL of RPMI 1640 medium. A positive and a negative control were both included and are displayed as polystyrene samples (PS) to validate the biofilm results (a positive control without a PEEK disk and a negative control without inoculum). Biofilm kinetics were established directly on the polystyrene 96-well plate. All plates were incubated at 37 °C for 72 h.

To observe changes in the biofilm stages, we used a destructive sampling design with time-series replicates. These time-series replicates corresponded to the adhesion, maturation, and dispersion stages of the biofilm. The cell suspension was removed from each well after adhesion (4 h). The wells were subsequently washed with sterile 1× PBS to remove non-adherent cells. For biofilm maturation and dispersion (24 and 48 h), the removed cell suspension was replaced with 200 µL of supplemented RPMI 1640 medium. Plates were kept incubated at 37 °C until the end of the experiment. After incubation, each time-series replicate was analyzed for biomass quantification, metabolic activity, and architectural description.

### 2.6. Quantification of C. albicans and N. glabratus Biofilm Biomass

The biofilm biomass was measured at 4, 24, and 48 h after crystal violet staining, using the protocol described by Christensen et al. [23] and modified by Ramírez Granillo et al. [24]. After supernatant removal (described above), the cells were fixed with 200 µL of 99% methanol for 15 min, then stained with 0.005% crystal violet for 20 min. Any excess stain was removed with distilled water, and the plates were air-dried at room temperature. The biomass was quantified after 15 min of solubilization with 200 µL of 33% (*v*/*v*) acetic acid (J.T. Baker^®^, Radnor, PA, USA). Absorbance was measured at 630 nm using an MRC UT-2100C microplate reader (MRC Ltd., Romeoville, IL, USA). Absorbance was directly proportional to the biofilm biomass.

### 2.7. Biofilm Metabolic Activity of C. albicans and N. glabratus

The MTS solution (Invitrogen^®^, Thermo Fisher Scientific, Waltham, MA, USA) was prepared by diluting the stock solution at a 1:10 (*v*/*v*) ratio. In vitro biofilm formation was performed as previously described. Following the incubation period, the supernatant was removed, and the samples were washed twice with 1× PBS. Subsequently, 2400 μL of 1× PBS and 480 μL of MTS solution were added, and the samples were incubated at 37 °C for 1 h in the dark. Finally, the supernatant was transferred to a 96-well microplate, and absorbance was measured at 490 nm using a T2100CMRC microplate reader (MRC Laboratory Instruments, Harlow, UK).

### 2.8. C. albicans and N. glabratus Biofilm Architecture Observation

After incubation, samples were fixed with 2.5% glutaraldehyde (Thermo Fisher Scientific, Waltham, MA, USA) for 3 h. Each disk sample was subjected to graded ethanol dehydration (10% to 90% ethanol, 10 min per concentration), followed by 30 min in absolute ethanol.

Samples were dried to the critical point using 1,1,1,3,3,3-hexamethyldisilazane (Sigma-Aldrich^®^, Allentown, PA, USA), sputter-coated with ionized gold at 5.0 kV and 10 µA, and observed under a Scanning Electron Microscope (JSM 7800 (Jeol Inc., Tokyo, Japan)) at the Scanning Microscopy Laboratory of the Center for Nanosciences, Micro- and Nanotechnologies, Instituto Politécnico Nacional (Mexico City, Mexico).

### 2.9. Physicochemical Characterization of Coated PEEK

The water contact angle measurements were performed using the sessile drop method. Droplets of 5 μL were gently deposited onto the sample surface using a micropipette. Images were acquired with a Fujifilm XT2 (Fujifilm^®^, Tokyo, Japan) digital camera equipped with a Tominon 135 mm f4.5 lens (Tomioka Optical, Tokyo, Japan), mounted on a Kopil Bellowsmat macro bellows extension (Kobayashi Seiki, Tokyo, Japan) and a set of 2 macro extension tubes measuring 11 mm and 16 mm. The camera and optics were mounted on a precision linear rail to achieve the desired magnification while maintaining a perpendicular optical axis. Diffused flash side lighting was used to obtain a well-defined droplet silhouette and minimize reflections at the solid–liquid interface. The camera was set in manual mode at the camera native lowest ISO 200, 1/125 shutter speed, f 11 and fixed focus throughout all measurements to ensure consistent image quality. Images were shot at a resolution of 6032 × 4032 pixels. Prior to analyses, all image samples were processed in Adobe Lightroom Classic to enhance exposition and contrast and improve edge definition without altering the droplet or surface geometry. Measurements were made by triplicate on Drop Analysis ver1.0.0rc4 (Computer software) retrieved from https://drop-analysis.com/ accessed on 1 July 2026 [25].

The morphology, particle size, and elemental composition of the TiO_2_ coatings were characterized by SEM coupled with energy-dispersive X-ray spectroscopy (EDS). Coated PEEK specimens were examined after colonization using a JSM 7800 (Jeol Inc., Tokyo, Japan) equipped with a bottom electron detector (LED), a backscattered electron detector (BED) and an energy-dispersive X-ray spectroscopy detector (EDS) to obtain the elemental microanalysis. Prior to analysis, the samples were prepared as described in Section 2.8. SEM micrographs were acquired at an accelerating voltage of 0.5 kV, a working distance of 4 mm, and magnifications ranging from 250× to 1000×. The morphology and distribution of the coating particles on the PEEK surface were evaluated from representative micrographs.

Particle size was determined by measuring more than 1000 particles. ImageJ^®^ software ver 1.54p was used to measure the particles. The images used for measurements were acquired using a backscattered electron detector. Each image was calibrated using its own scale, and automated measurements were performed. Filters for circularity, minimum size, and maximum size were applied during measurements. The data were organized into 10 intervals. For the grouped data, the lower limit was 42 nm and the upper limit was 250 nm, resulting in a class width of 20 nm. Class frequencies were tallied.

Elemental characterization was performed by EDS analysis of the representative regions of the coated surfaces. EDS spectra were acquired under the following conditions: an accelerating voltage of 20 kV and a working distance of 10 mm. Multiple regions of each coating were analyzed to account for spatial variation in elemental composition. The EDAX Genesis Software (AMETEK, Berwyn, PA, USA) was used for EDS. Backscattered electron (BE) data collection and material composition analysis were performed via microanalysis techniques. The SEM-EDS analysis was used to confirm the presence and distribution of Ti and other elements associated with the coating and substrate.

## 3. Results and Discussion

**Figure 1:**
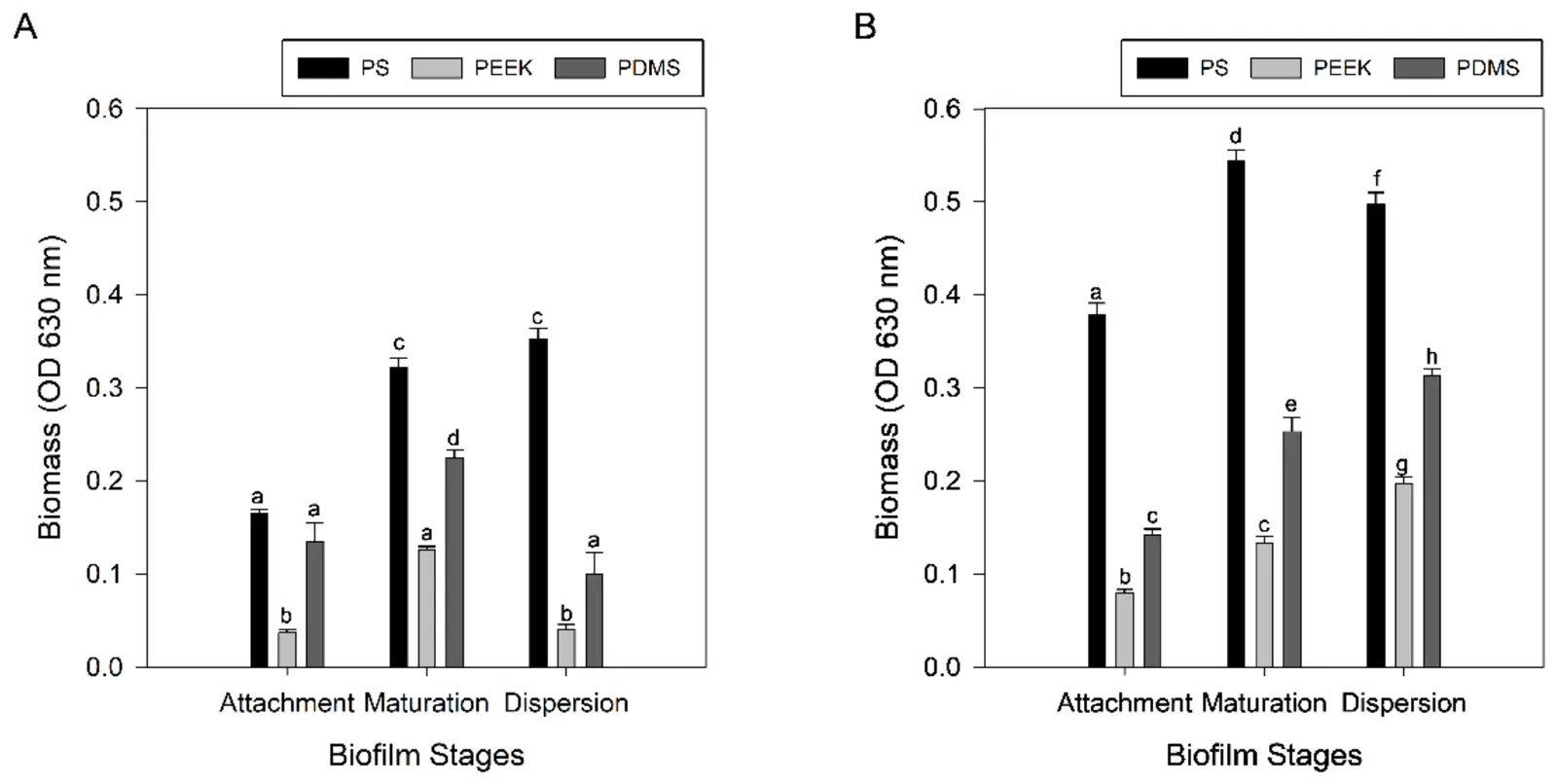
Adhered biomass of (**A**) *C. albicans* and (**B**) *N. glabratus* on polystyrene—PS, polyetheretherketone—PEEK, and polydimethylsiloxane—PDMS disks at different stages of biofilm development. Different letters above the bars indicate significant differences, which was set at *p* < 0.05. Error bars belong to one standard deviation ± the mean.

Biofilm biomass varied significantly across materials and fungal species. Compared to the positive control in polystyrene (PS), both biomaterials exhibited a reduced biomass of each fungus during the maturation and dispersion stages, with PEEK consistently being the one with the lowest biomass across all stages (Figure 1). Although both strains behaved similarly between PEEK and PDMS, the reduction in biofilm biomass resulted significantly higher for *N. glabratus*, as this fungus possesses a greater capacity for biofilm formation on PS (Figure 1B). According to different studies, neither PEEK nor PDMS possess any inherently antimicrobial properties [26,27,28]. However, some structural features, like surface roughness, free energy, and hydrophobicity, may positively or negatively affect microbial adhesion and subsequently biofilm formation, although complete inhibition must be difficult to achieve without any antimicrobial additives or modifications [29].

**Figure 2:**
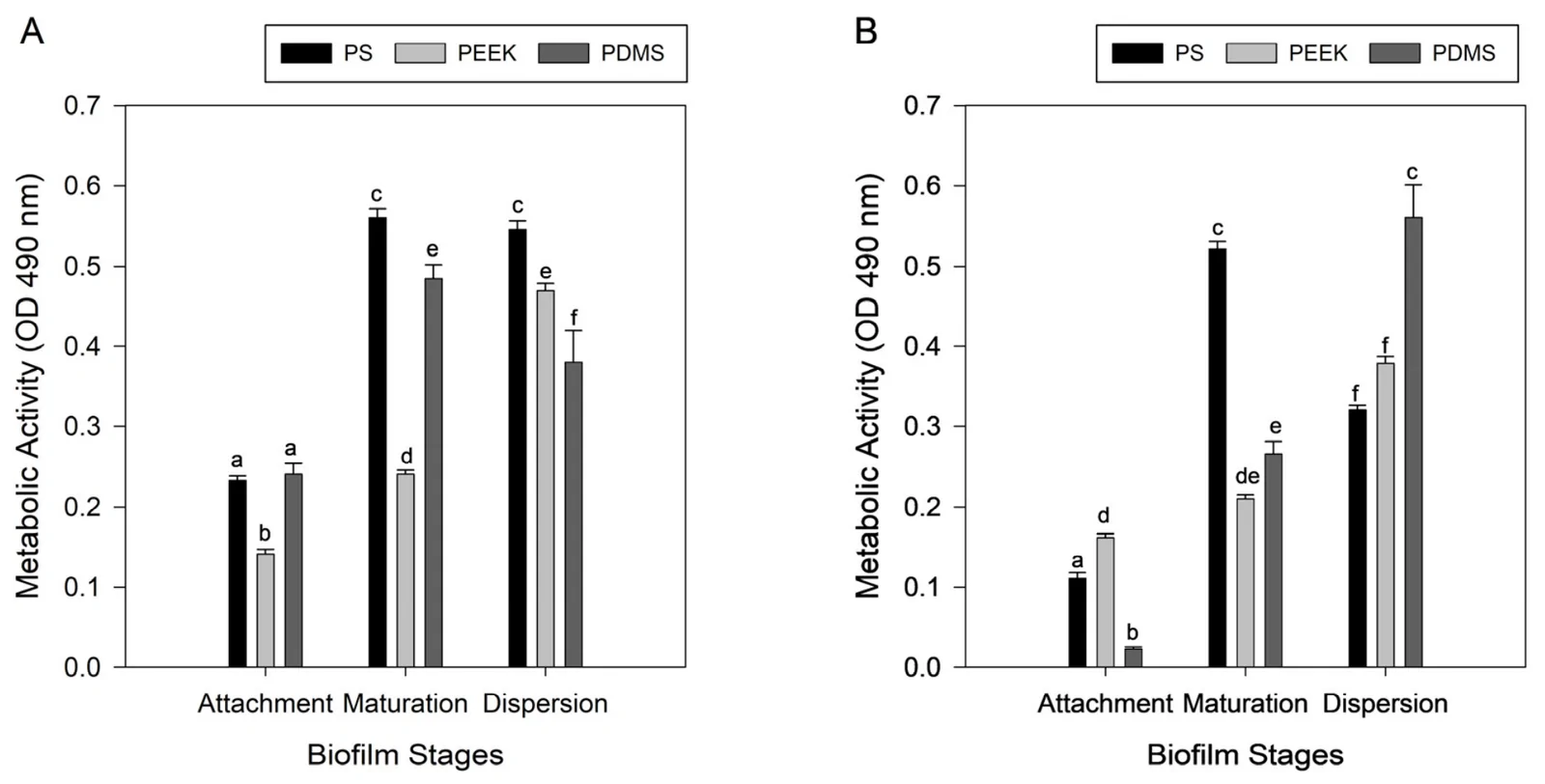
Metabolic activity of in vitro biofilms formed by (**A**) *C. albicans* and (**B**) *N. glabratus* on polystyrene—PS, polyether etherketone—PEEK, and polydimethylsiloxane—PDMS disks at different stages of biofilm development. Different letters above the bars indicate significant differences, which was set at *p* < 0.05. Error bars belong to one standard deviation ± the mean.

Indeed, the cells successfully attached to their surfaces were highly viable and metabolically active (Figure 1), although fewer attached cells and less EPS accumulation on biomaterial disks were detected (Figure 2). Biofilm metabolism presented a different dynamic according to each development stage, as this was reduced during maturation but recorded more activity than the control during dispersion. The differences in biofilm biomass on PEEK and PDMS indicated an alteration at the attachment stage and in matrix accumulation rather than a complete suppression of fungal metabolic activity. Biofilm biomass and metabolic activity are interrelated but may respond differently depending on environmental or surface conditions [30].

Overall, *N. glabratus* exhibited significantly lower metabolic activity on all surfaces throughout biofilm development, suggesting a reduced capacity for biofilm maturation and/or lower physiological activity under the tested conditions (Figure 2B). In contrast, *C. albicans* maintained similar metabolic activity on polystyrene (PS) and the evaluated biomaterials, indicating that these surfaces did not substantially impair its biofilm-associated viability or metabolic adaptation (Figure 2A). These results may indicate the greater biofilm-forming versatility and surface adaptability commonly associated with *C. albicans.* Some structural features of *C. albicans* influence the complexity of its biofilm architecture, with true hyphae formation being one of the most important factors, whereas *N. glabratus* presents a thinner and less complex ultrastructure [31,32]. More structurally complex biofilms are generally associated with increased resilience and persistence [33].

**Figure 3:**
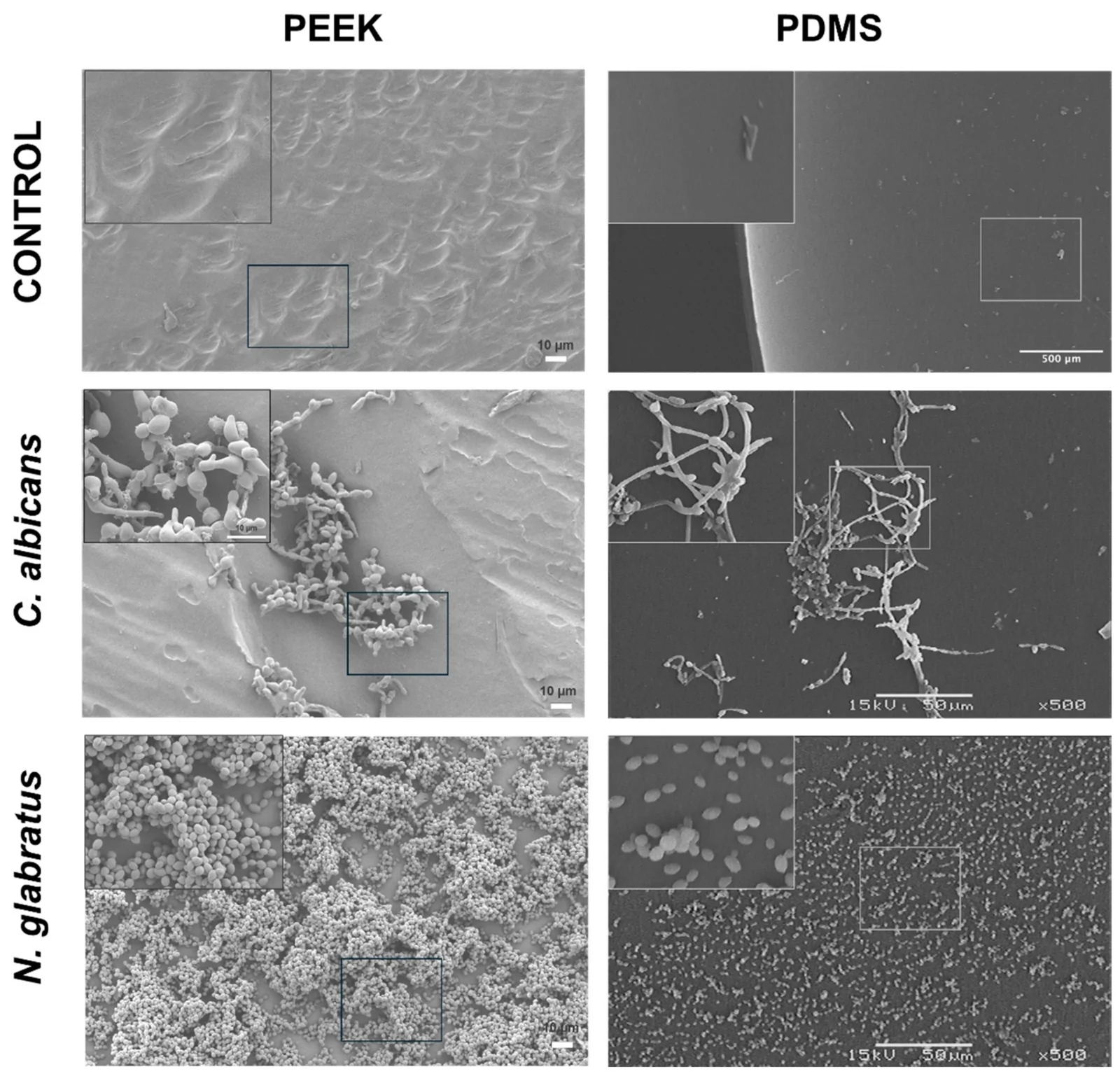
Architectures of *C. albicans* and *N. glabratus* matured biofilms (24 h incubated) on uncoated biomaterial surfaces (polyether etherketone, PEEK; polydimethylsiloxane, PDMS).

Scanning electron microscopy (SEM) confirmed the species-specific adhesion and subsequent biofilm formation on PEEK and PDMS (Figure 3). On one hand, control surfaces showed no microbial structures and displayed the characteristic morphology of each material. Inoculated samples, on the other hand, demonstrated clear evidence of microbial proliferation of both species. Although these architectures do not necessarily correspond to a mature biofilm, cell aggregation and the presence of filamentous forms on the biomaterial surfaces were observed.

As shown in the control panel, PEEK disks presented more roughness and some irregularities on their surface. The latter provided anchoring points for adhesion; therefore, a more structured biofilm with greater surface coverage was observed with PEEK. In contrast, PDMS’s cell density was minor and more disperse. These findings indicate that the biomaterial composition influences fungal attachment behavior and biofilm spatial organization.

During biofilm maturation, *C. albicans* exhibited fewer attached cells on PEEK and PDMS surfaces compared to *N. glabratus*. This may reflect species-specific biofilm organization strategies, since *C. albicans* typically develops more complex three-dimensional biofilms characterized by hyphal formation and vertical structuring [34], whereas *N. glabratus* forms more compact yeast-based biofilms with strong surface-associated adhesion [35].

The selection of PEEK as the substrate for coating deposition was based on methodological considerations. The TiO_2_/ZrO_2_ coatings were produced using spin-coating procedures, which are more readily applied to rigid substrates, such as PEEK. In contrast, PDMS presents additional challenges related to its surface chemistry, low surface energy, hydrophobicity, and elastic properties, which can compromise coating adhesion, uniformity, and reproducibility [36]. Therefore, the development and optimization of TiO_2_/ZrO_2_ coatings on PDMS surfaces were considered beyond the scope of the present study and will be addressed in future work.

**Figure 4:**
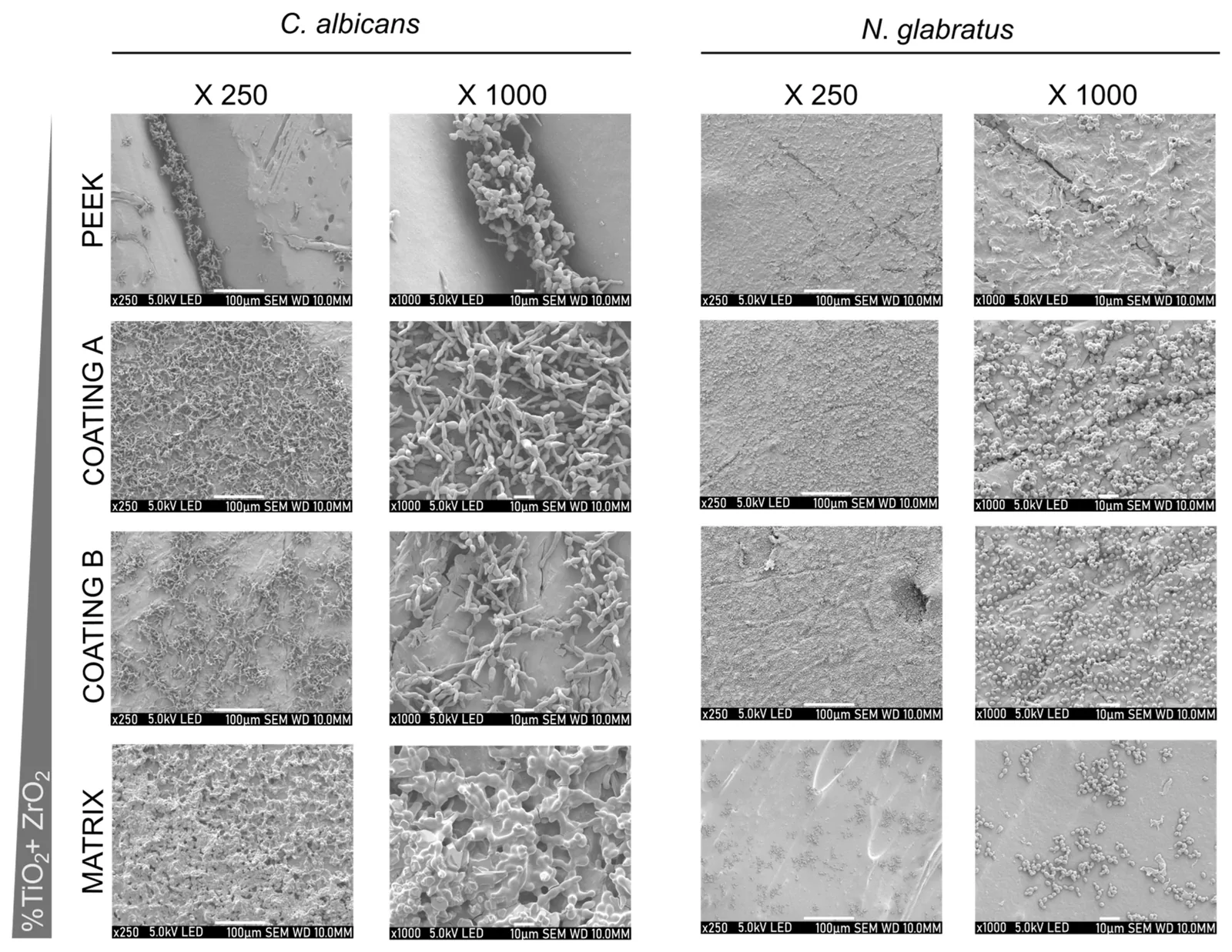
Architectures of *C. albicans* and *N. glabratus* matured biofilms (24 h incubated) on coated PEEK surfaces at 250 and 1000×.

In Figure 4, the mature *C. albicans* biofilms are displayed for each biomaterial tested, including the sol–gel matrix control. In the sol–gel micrography, we observed a very-well-structured mature biofilm, where blastoconidia were completely embedded within the extracellular matrix and mature structures, like water-filled channels, were evidenced. However, less established biofilms were shown in coating A and coating B samples, where a Ti signal was confirmed by EDS. A clear inhibition of the *C. albicans* biofilm was only observed under coating B’s application. In this panel, the biofilm was less condensed, while neither EPS nor other structures were shown.

In general, extra layers added by any kind of coatings modified the materials’ surfaces either structurally and/or biochemically [37]. Biochemically, TiO_2_/ZrO_2_ coatings disrupted EPS and extracellular matrix formation (as seen in Figure 4) and reduced biofilm biomass (only with coating B) by inducing oxygen radicals, which decreased cellular antioxidants and overproduced superoxide ions [38,39]. Although these mechanisms were not directly evaluated in the present study, they could potentially contribute to the reduced extracellular matrix and less condensed biofilm architecture observed for coating B. Therefore, the antibiofilm response observed here may result from a combination of altered surface properties (as discussed below) and coating-associated biological effects.

*N. glabratus* formed mature biofilms indifferently in the PEEK, sol–gel matrix, and coating A samples. All of these showed a dense aggregate of blastoconidia, extracellular matrix covering, and signals of water-filled channels. Compared to these treatments, coating B’s disks inhibited biofilm maturation (only planktonic cells were observed attached to the disk surface). Thus, the surface modifications that occurred due to sol–gel matrix and coating A were insufficient to prevent biofilm maturation, whereas coating B interfered with biofilms development after initial attachment.

Coating B comprised a thicker layer of titanium; together they have been associated with antimicrobial and anti-adhesive effects as discussed above. However, *N. glabratus* exhibited a stronger inhibitory response to coating B than *C. albicans.* Intrinsic biological differences between the species were again exposed, highlighting the developed biofilm-forming capacity of *C. albicans* [40].

In conclusion, the inhibitory effect observed with both strains by coating B successfully interfered with maturation events and extracellular matrix establishment. However, the difference between these effects indicated potential species-specific antibiofilm activity of TiO_2_/ZrO_2_ coatings on PEEK materials.

**Figure 5:**
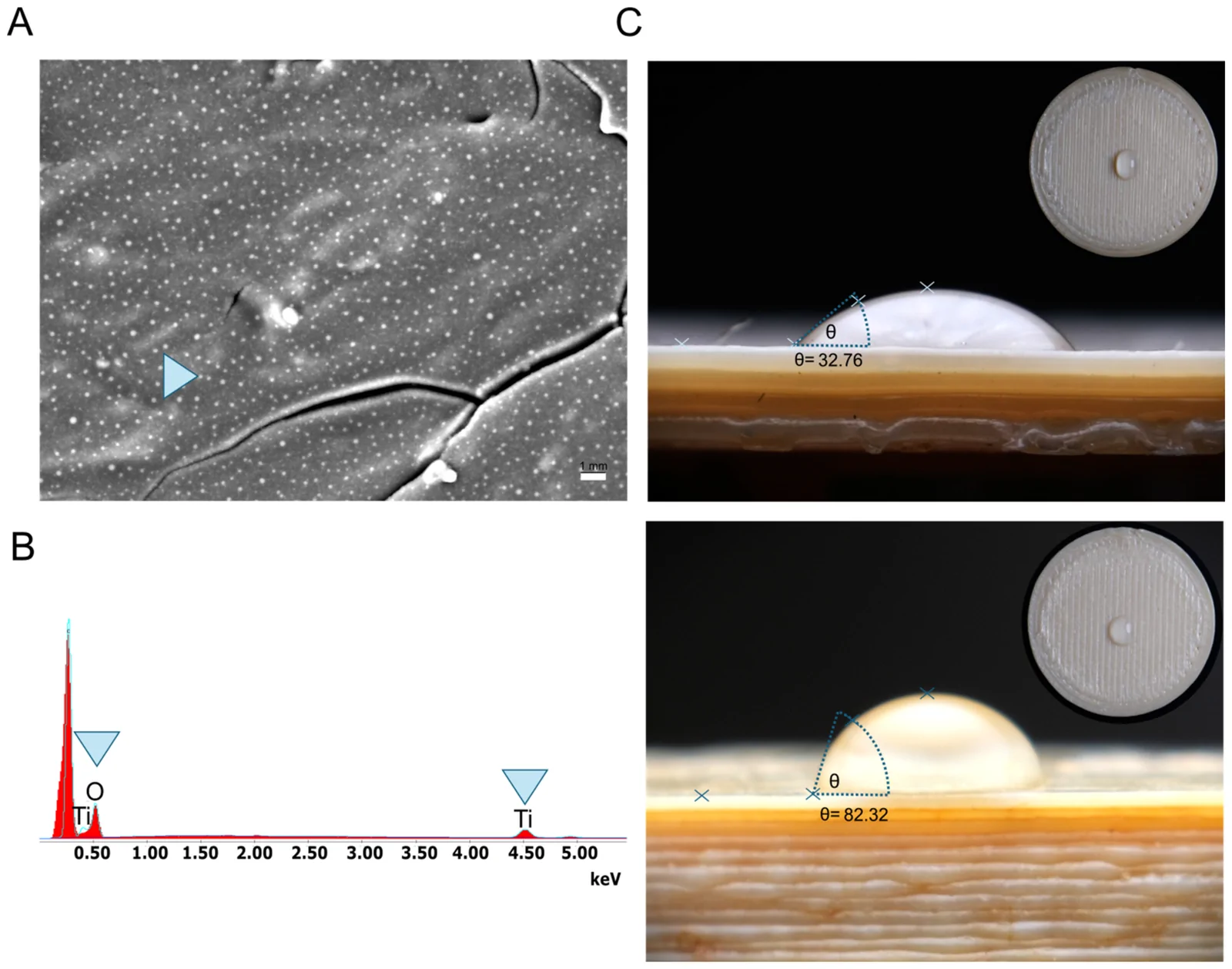
Physicochemical characterization of TiO_2_/ZrO_2_-coated PEEK disks—coating B compared to an uncoated PEEK. (**A**) SEM morphology of coating B’s surface, (**B**) EDS spectrum, and (**C**) water contact angle measurements. Blue inverted triangles highlight the exact place were the EDS was directed, indicating the main elements of the specific particle. “X” symbols are the identification points for the water contact angle (θ) measurement. The θ corresponds to the average water contact angle considering n=3 replicates drop and material samples.

Due to the greatest reduction in biofilm development shown by coating B’s treatment, this was further characterized to identify physicochemical features that could contribute to the observed response. As shown in Figure 5, coating B presented a distinct morphological and physicochemical features compared to the control (uncoated PEEK). SEM analysis revealed a homogeneous distribution of titanium nanoparticles with a mean size of 133 nm and a standard deviation of 55 nm. The particle-size distribution appears to consist of two populations: one centered near 50 nm and another near 150 nm. The overall average size was 133 nm with a standard deviation of 50 nm (Figure 5A). EDS confirmed the presence of titanium in the analyzed coated region. In addition, water contact angle measurements indicated a significant decrease in wettability relative to uncoated PEEK, indicating a transition from a hydrophilic (θ < 30°) to a hydrophobic (90° < θ < 120°) surface on coated PEEK disks.

Surface characteristics, such as porosity, roughness, wettability, morphology, and anisotropy, have been proven to have an impact on the initial cell adhesion; more importantly, the increment of the surface contact area could be critical in determining how and where microbes adhere to the surfaces [33]. In the present study, the SEM morphology of coating B suggests that the coating formed a relatively uniform nanoparticulate layer over the PEEK surface. Such a layer could partially modify the underlying surface topography, including the microscopic irregularities characteristic of uncoated PEEK [41]. Surface wettability is an important physicochemical property influencing the interaction between microorganisms and biomaterial surfaces. Changes in the contact angle can modify the balance of hydrophobic and hydrophilic interactions between microbial cells and the substrate, thereby affecting the probability and strength of initial adhesion [42]. However, the relationship between surface hydrophobicity and microbial adhesion is not universal and may depend on the physicochemical properties of both the microorganism and the material, as well as the composition of the surrounding medium [43]. The association observed between the results presented for coating B and both fungal species’ biofilm formation suggested that the modified surface properties may have contributed to the observed biofilm response, particularly during the early stages of surface colonization. Nevertheless, because adhesion was not directly quantified in relation to the contact angle and no mechanistic assay was performed, the contribution of wettability cannot be established as the sole mechanism underlying the antibiofilm effect.

## 4. Conclusions

Both PEEK and PDMS support biofilm formation by *C. albicans* and *N. glabratus*, although differences in the biomass and biofilm architecture were observed between the biomaterials. In particular, PEEK exhibited a greater resistance to dense biofilm formation, whereas the remaining metabolic activity of the biofilms was not completely suppressed. Among the evaluated surface modifications, coating B produced the greatest reduction in biofilm biomass and maturation for both fungal species, especially of *C. albicans*. The TiO_2_/ZrO_2_-coating treatment (coating B) produced a significant modification of PEEK’s surface wettability, apparent roughness, and uniformity. These physicochemical changes may contribute to the observed differences in fungal colonization and biofilm development, although their specific contribution requires further investigation. Overall, the findings support the potential of Ti- and Zr-based surface modification as an approach for reducing fungal biofilm development with PEEK, while further studies are needed to clarify the underlying mechanisms and optimize the coating performance.

## Data Availability

The original contributions presented in this study are included in the article. Further inquiries can be directed to the corresponding authors.
